# Bisphosphonate‐Induced Deterioration of Osteomalacia in Undiagnosed Adult Fanconi Syndrome

**DOI:** 10.1002/jbm4.10374

**Published:** 2020-06-05

**Authors:** Tim Cundy, Lorna Que, Ibrahim M Hassan, Louise Hughes

**Affiliations:** ^1^ Department of Endocrinology Greenlane Clinical Centre Auckland New Zealand; ^2^ Nuclear Medicine, Department of Radiology Auckland City Hospital Auckland New Zealand; ^3^ Department of Anatomical Pathology Concord Hospital Sydney Australia

**Keywords:** ANALYSIS/QUANTITATION OF BONE, BONE HISTOMORPHOMETRY, BONE SCINTIGRAPHY, DISEASES AND DISORDERS OF/RELATED TO BONE, OSTEOMALACIA AND RICKETS, THERAPEUTICS, ANTIRESORPTIVES

## Abstract

We describe two women with a misdiagnosed fracturing bone disease who were treated erroneously with i.v. zoledronate. Over the next year, they suffered marked clinical and radiographic deterioration in skeletal disease. Both were eventually diagnosed with hypophosphatemic osteomalacia secondary to acquired Fanconi syndrome (caused by light‐chain myeloma in one case and tenofovir treatment in the other). Appropriate treatment with phosphate supplementation was instituted with clinical improvement. These cases illustrate the importance of not missing osteomalacia in adults presenting with fractures, and the potentially damaging effects of treatment with long‐acting inhibitors of bone resorption in these circumstances. © 2020 The Authors. *JBMR Plus* published by Wiley Periodicals, Inc. on behalf of American Society for Bone and Mineral Research.

## Introduction

Osteomalacia is a bone disease of adults that reflects generalized impairment of osteoid mineralization. It can manifest clinically with bone pain and fractures, and may sometimes be mistaken for osteoporosis, which is much more prevalent.^(^ ^1^ ^)^ Potent i.v. bisphosphonates are widely used to treat osteoporosis. They act chiefly through the inhibition of bone resorption, but unlike the prototype bisphosphonate, etidronate, they do not themselves cause defective mineralization.^(^ ^2^ ^)^ A number of case reports have described adults erroneously given bisphosphonates^(^ ^3^, ^4^, ^5^, ^6^ ^)^ or denosumab^(^ ^7^ ^)^ for what was subsequently thought to be osteomalacia. In these reports, emphasis had been given on the biochemical effects—hypocalcemia and hypophosphatemia—induced by a rapid reduction in bone resorption. Apart from a report of one patient who had increased disability and weakness,^(^ ^4^ ^)^ there has been little to suggest adverse skeletal consequences, and none have reported histological findings.

We describe two women with a fracturing bone disease who were treated with i.v. zoledronate and suffered marked clinical deterioration in skeletal disease. Both were eventually diagnosed with hypophosphatemic osteomalacia secondary to acquired Fanconi syndrome, and appropriate treatment was instituted with clinical improvement. These cases illustrate the importance of not missing osteomalacia, with or without fractures. Treatment with inhibitors of bone resorption in this circumstance is probably damaging.

## Case Reports

### Subject A

**Figure 1:**
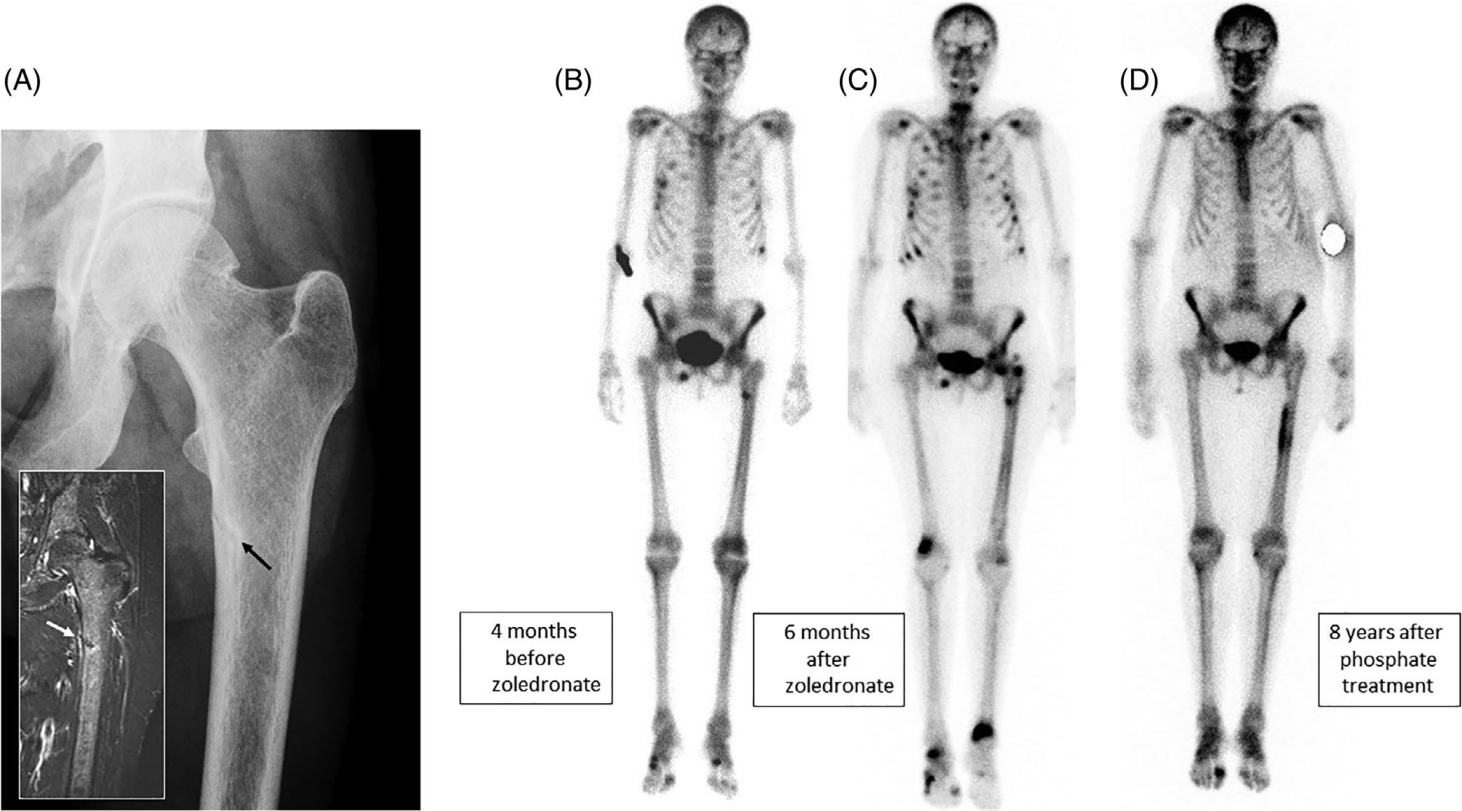
Radiographic images from subject A. (*A*) Looser zone (pseudofracture) of the upper femur (inset T2‐STIR MR image) that occurred 4 months before zoledronate was administered. This was originally interpreted as a “stress fracture.” (*B*) Bone scintigram taken at the same time as the images shown in *A*. There is increased uptake of isotope at the site of the Looser zone, but also in several ribs and the right inferior pubic ramus. (*C*) Bone scintigram taken 6 months after the first zoledronate infusion. There are numerous new lesions in the ribs, both femora and the distal tibias. (*D*) Bone scintigram taken 8 years after phosphate treatment was started. There is increased uptake in relation to the left femoral rod and degenerative disease in the feet, but the other lesions have healed.

Subject A began to experience bone pain and difficulty mobilizing at the age of 50. Radiographs showed metatarsal, rib, and pelvic fractures. Four years earlier she had been diagnosed with localized breast cancer and had been treated with surgery and radiotherapy, and then tamoxifen. The fractures began around the time tamoxifen was discontinued. Radiographs showed right‐sided pubic rami fractures that were interpreted as metastases. Her plasma calcium was 2.29 mmol/L and phosphate 0.89 mmol/L (normal ranges 2.2 to 2.6 and 0.8 to 1.4 mmol/L, respectively). The alkaline phosphatase (ALP) was noted to be elevated (162 U/L), and attributed to the fractures. Vitamin D treatment was prescribed and palliative radiotherapy to the pubic rami was administered. Two months later she suffered what was described as a “stress fracture” of the proximal femur (Fig. 1*A*), which was managed with an intramedullary nail. Decalcified bone reamings from this procedure showed no evidence of metastatic disease. She continued to suffer pain and increasing difficulty mobilizing. Her bone density was normal, but a scintigram (Fig. 1*B*) showed several skeletal lesions. At this time, her plasma calcium was 2.17 mmol/L, phosphate 0.70 mmol/L, and ALP 180 U/L. Intravenous zoledronate (4 mg) was administered on the assumption that the lesions indicated metastatic breast cancer. Her clinical symptoms deteriorated and a new bone scintigram showed numerous new fractures (Fig. 1*C*). A second infusion of zoledronate was administered 12 months after the first, with subsequent worsening of symptoms. Plasma calcium and phosphate levels were not recorded before this infusion, but her ALP was 106 U/L. No investigations to exclude myeloma had been undertaken.

**Figure 2:**
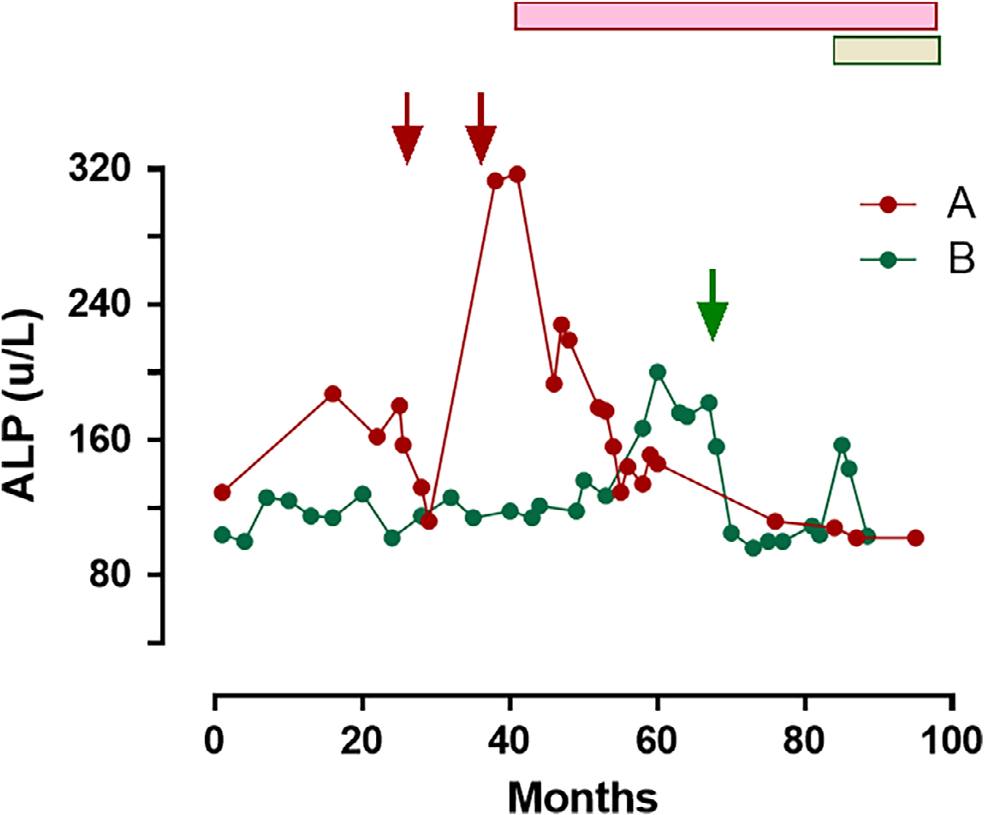
Serial changes in alkaline phosphatase activity in subjects A (red) and B (green). Solid arrows above indicate timing of zoledronate infusions. The horizontal bars indicate the time on phosphate replacement therapy (48 mmol/day of elemental phosphorus in both subjects). ALP = alkaline phosphatase.

**Figure 3:**
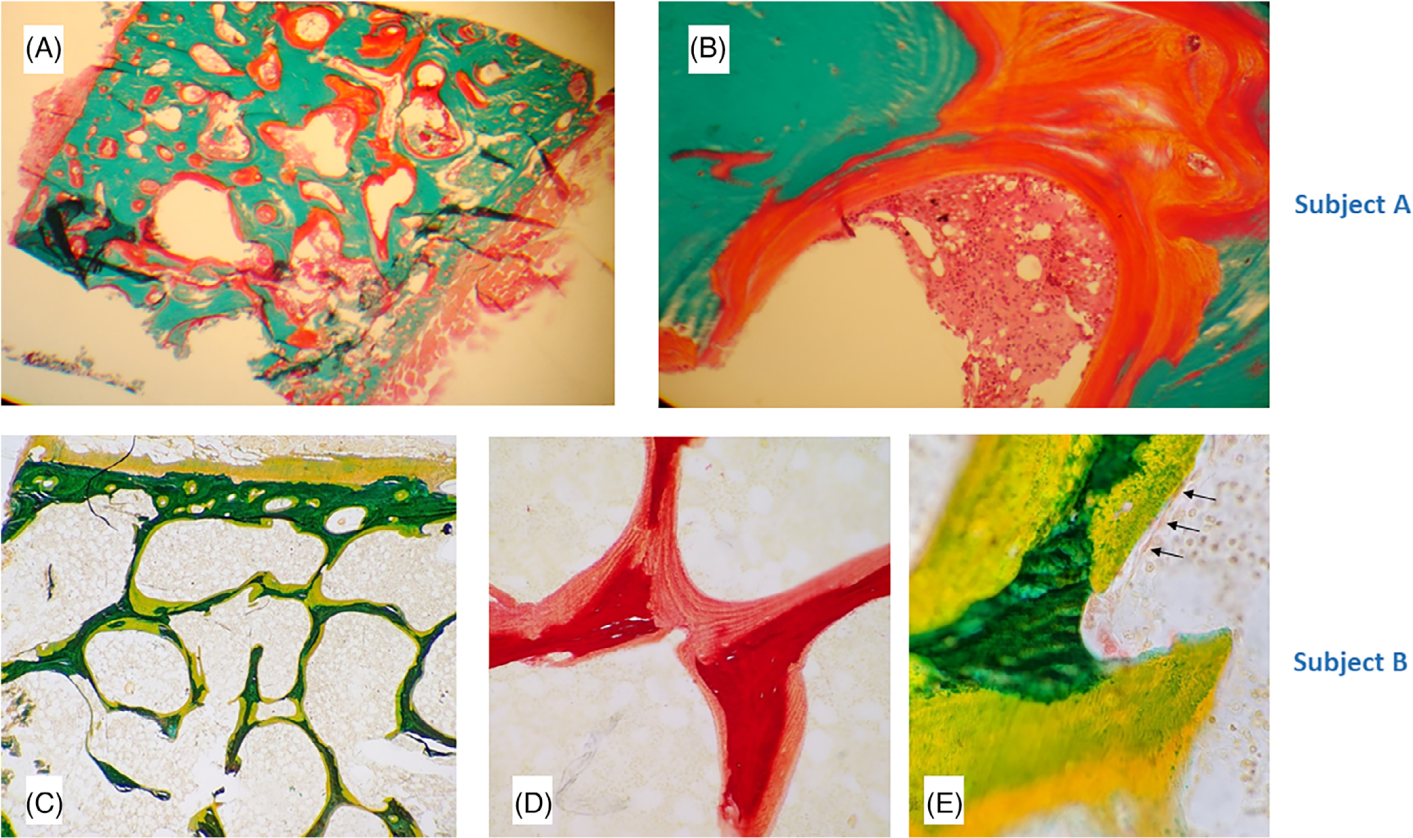
Bone biopsy findings. Subject A = upper panels. (*A*) Goldner trichrome stain (×20) in which mineralized bone appears green and unmineralized osteoid orange. All bone surfaces are covered in a thick layer of osteoid up to 13 lamellae thick, signifying severe osteomalacia. (*B*) (×200) Very few osteoblasts can be seen on the osteoid surface. Subject B = lower panels. (*C*) Alkaline phosphatase stain (×40) in which mineralized bone appears green and unmineralized osteoid yellow. All bone surfaces are covered in osteoid. (*D*) Sirius red stain (×200) showing osteoid (pink) that is up to 9 lamellae thick, signifying severe osteomalacia. (*E*) Alkaline phosphatase stain (×600) showing flat inactive osteoblasts on the osteoid surface (arrows).

A review of her case in our service 14 months after her initial presentation found that her plasma phosphate had been 0.70 mmol/L at the time of her fractures. A month after the first zoledronate treatment the plasma phosphate had fallen to 0.43 mmol/L, the urine N‐telopeptide (a bone resorption marker) had fallen from 170 to 55 nmol BCE/mmol creatinine (NR 14–74), and there had been a transient reduction in ALP (Fig. 2). A tetracycline‐labeled transiliac bone biopsy (undecalcified) showed florid osteomalacia with most bone surfaces covered with osteoid and relatively few osteoblasts. Under polarized light, there were up to 13 osteoid lamellae (normal ≤4) and no uptake of the tetracycline label was visible (Fig. 3*A* and *B*).

Further investigation confirmed the diagnosis of Fanconi syndrome with aminoaciduria, renal phosphate and glucose loss, metabolic acidosis, and increased urine β2‐microglobulin excretion (Table 1) caused by κ‐light chain myeloma. Phosphate supplementation (phosphate; Sandoz, Holzkirchen, Germany) was begun with 1.5 g (48 mmol) a day in divided doses of elemental phosphorous. The treatment was well‐tolerated. Her bone pain resolved, and there were no more fractures. A scintigram 8 years later showed healing of the skeletal lesions (Fig. 1*D*). The myeloma subsequently relapsed and she had a successful autologous bone marrow transplant. Assessment of her renal tubular function a year after transplantation showed that the Fanconi syndrome had not resolved.

**Table 1:** Laboratory Findings at Time of Diagnosis of Fanconi Syndrome

	Case 1	Case 2	Normal values
Cause of Fanconi syndrome	Light chain myeloma	Tenofovir treatment	‐
Gender/age	F 52	F 69	‐
Calcium (mmol/L)	2.13	2.27	2.2–2.6
Phosphate (mmol/L)	0.44	0.37	0.8–1.4
Alkaline phosphatase^a^ (u/L)	157	174	40–120
Plasma calcidiol (nmol/L)	107	137	50–150
TmP/GFR (mmol/L GF)	0.5	0.3	0.8–1.4
Estimated GFR (mL/min)	52	50	>90
Glucose/creatinine clearance (%)	10	8	<1
Urine β2‐microglobulin (μg/L)	73.5	72.7	<5
Urine aminoaciduria	Marked, generalized	Not tested	None
Plasma bicarbonate (mmol/L)	20	19	23–30
BMD lumbar spine *T*‐score	−0.9	−1.8	−2 to +2

^a^ Before zoledronate treatment.

GFR = Glomerular filtration rate; TmP = Tubular maximum rate of phosphate reabsorption relative to glomerular filtration ratea.

### Subject B

**Figure 4:**
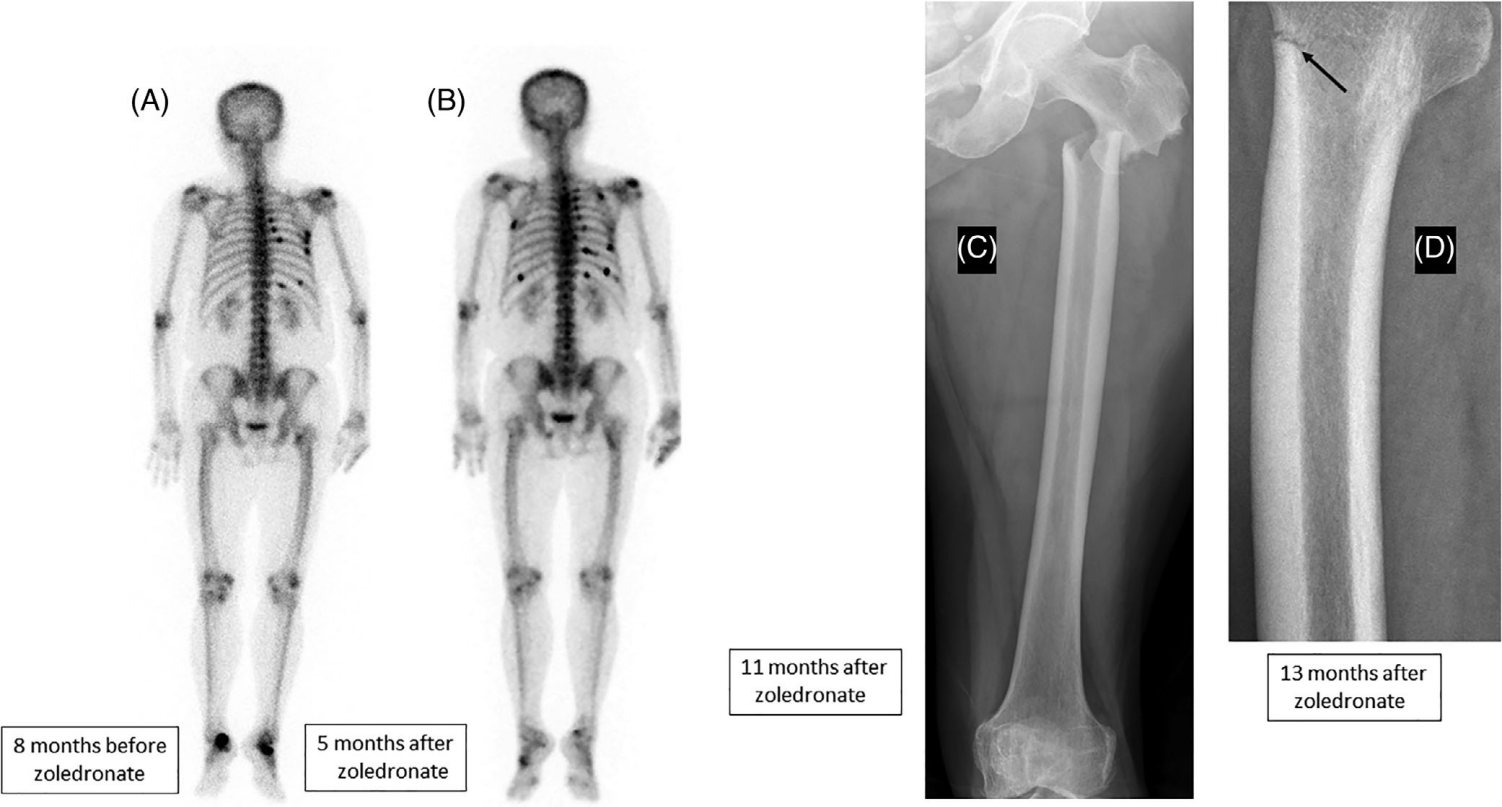
Radiographic images from subject B. (*A*) Bone scintigram (posterior view) taken 8 months before zoledronate treatment showing rib fractures. (*B*) Bone scintigram taken 5 months after the zoledronate infusion. There are several new rib lesions and increased uptake in the left proximal femur. (*C*) Fracture of the left upper femur 11 months after the zoledronate infusion. (*D*) Looser zone (pseudofracture) the right upper femur that occurred 2 months later.

Subject B had e‐antigen‐positive chronic hepatitis B, with steatosis on her liver biopsy. Treatment with lamivudine was started at age 61. Her HBV genotype indicated lamivudine resistance, so treatment was changed to tenofovir 2 years later. Plasma calcium and phosphate concentrations were not measured at this time, but her ALP was 108 U/L. At age 68, she began to complain of chest pain and a scintigram identified a number of rib fractures (Fig. 4*A*). Her ALP was elevated (174 u/L) with other liver enzymes normal, and this was attributed to the rib fractures. Her plasma calcium was 2.17 mmol/L and her phosphate was 0.70 mmol/L. Serum protein electrophoresis was normal. Vitamin D treatment was prescribed. A bone density scan indicated spinal osteopenia (Table 1). Intravenous zoledronate (5 mg) was administered, but she experienced an increase in rib pain. A second scintigram suggested several new fractures and increased isotope uptake in the upper left femur (Fig. 4*B*). Eleven months after receiving zoledronate she fractured her left femur, and 2 months later her right femur (Fig. 4*C*,*D*).

A review of her case in our service found that since taking tenofovir she had had persistent mild hypophosphatemia (0.8 to 0.9 mmol/L). A month after zoledronate treatment the plasma phosphate had fallen to 0.66 mmol/L, and there had been a transient reduction in ALP (Fig. 2). A single tetracycline‐labeled transiliac bone biopsy (undecalcified) showed severe osteomalacia. Most bone surfaces were covered by thick osteoid, and there were few active osteoblasts (Fig. 3 *C‐E*). Up to nine osteoid lamellae were seen (normal ≤4), and only minimal blurred tetracycline labeling was identified.

Further investigation confirmed the diagnosis of Fanconi syndrome with renal phosphate and glucose loss, metabolic acidosis, and increased urine β2‐microglobulin excretion (Table 1), likely caused by tenofovir. The treatment for hepatitis B was changed to entecavir and a phosphate supplement (Sandoz) was begun providing 1.5 g (48 mmol) a day in divided doses, of elemental phosphorous. The treatment was well‐tolerated. Her bone pain and mobility improved over the next few months, and there were no further fractures. Assessment of her renal tubular function 6 months after stopping tenofovir showed that the Fanconi syndrome had resolved.

## Discussion

We describe the diagnosis of symptomatic osteomalacia in two cases resulting from chronic hypophosphatemia that was overlooked, being mistaken for metastatic breast cancer in subject A and osteoporosis in subject B. Zoledronate, a long‐lasting and potent inhibitor of bone resorption, was administered to both. Early biochemical changes were seen—a reduction in bone turnover and a fall in the already low plasma phosphate levels—and within 6 months both subjects experienced marked clinical deterioration with new scintigraphic lesions (Figs. 1*B*,*C* and 4*A*,*B*) and later, new fractures (Fig. 4*C*,*D*).

Osteomalacia is a disorder characterized by defective mineralization of newly formed osteoid. Broadly speaking, there are three main causes. In calcipenic osteomalacia, there is inadequate calcium available for mineralization. In phosphopenic osteomalacia, chronically low phosphate levels limit mineralization, and osteoblast dysfunction where despite adequate mineral availability, it cannot be utilized (hypophosphatasia is the most frequent cause in this category). Adult physicians not uncommonly encounter calcipenic osteomalacia in the context of malnutrition, malabsorption, and vitamin D deficiency, particularly in the institutionalized elderly. The other forms are distinctly rarer, and unfamiliarity with them was probably a factor in the diagnosis being overlooked in the cases we described here. There are numerous genetic causes of phosphopenic osteomalacia, but these present, and are usually detected, in childhood. In adults, the most frequently encountered causes of acquired phosphopenic osteomalacia are oncogenic osteomalacia (caused by ectopic FGF23 production), repeated i.v. iron administration,^(8^ ^)^ and Fanconi syndrome—a generalized proximal renal tubular dysfunction characterized by urinary loss of glucose, phosphate, amino acids, and β2‐microglobulin, as well as a reduction in acid clearance. Acquired Fanconi syndrome in adults can be caused by amyloidosis, heavy metal toxicity (including lead, cadmium, and mercury), drugs (including cisplatin, ifosfamide, tenofovir, adefovir, cidofovir, valproate, and aminoglycoside antibiotics),^(^ ^9^ ^)^ or by myeloma from the deposition of light chains in the renal tubules.

In retrospect, a number of important clues had been overlooked in the two patients referred to us. These included the misattribution of bone pain and elevated ALP values to fractures, and Looser zones or pseudofractures in the upper femur—a hallmark of osteomalacia—incorrectly interpreted as stress fractures. There was clearly a “framing” bias in the case of subject A, whose diagnosis of breast cancer 4 years earlier influenced a number of incorrect diagnostic interpretations and actions. The skeletal scintigrams were highly suggestive of osteomalacia. Skeletal scintigraphy uses a radiolabeled bisphosphonate (^99^Tc‐methylene diphosphonate) that is taken up at mineralizing surfaces and provides information not only the anatomic location of lesions, but also on bone pathophysiology. In osteomalacia, the scintiscan typically shows a high degree of isotope uptake, particularly in the skull and lower limbs,^10^, ^11^ ^)^ a feature seen in both our cases. Skeletal uptake can be so high that little isotope is excreted and the kidneys are poorly visible. The numerous transverse rib lesions in subject A were also typical of fractures in osteomalacia; with bone metastases, the scintigraphic lesions tend to follow the axis of the ribs. Plasma phosphate concentrations at the lower end of the normal range were also overlooked, possibly because of underappreciation of its normal diurnal variation. Samples drawn in the late morning or afternoon may be up to 0.4 mmol/L higher than those drawn at the early morning nadir.^(^ ^12^ ^)^

As mentioned above, there are a small number of case reports describing the effects of bisphosphonates or denosumab mistakenly given to adults with presumed osteomalacia.^(^ ^3^, ^4^, ^5^, ^6^, ^7^ ^)^ These reports have been concerned largely with biochemical changes in calcium, phosphate, PTH, or ALP, with little comment on adverse clinical outcomes (Table 2). None have described bone histology. In both subjects A and B, there was a decline in plasma phosphate after zoledronate was given, probably related to a reduction in bone resorption and secondary hyperparathyroidism. The bone histology findings were striking with bone surfaces covered with large volumes of osteoid, but low osteoblast numbers and almost no tetracycline label visible under polarized light. Our interpretation is that on top of pre‐existing osteomalacia, zoledronate treatment had “frozen” cellular activity so that bone self‐repair was severely impaired, precipitating the increased fracture rate. We described recently a similar case in an adult with chronic renal failure, whose previously asymptomatic hypophosphatasia changed—with the development of multiple fractures—after he was prescribed alendronate.^(^ ^13^ ^)^

**Table 2:** Previously Reported Cases of the Effects of Erroneous Administration of Potent Inhibitors of Bone Resorption in People With Osteomalacia

Reference	2	3	4	5	6
Age/gender	52 F	42 F	71 F	54 F	48 M
Cause of osteomalacia	Vitamin D deficiency	Vitamin D deficiency	Oncogenic osteomalacia	Oncogenic osteomalacia	Fanconi syndrome
Antiresorptive treatment	Pamidronate 60 mg ×1	Alendronate 1 year ‐ oral	Zoledronate 4 mg ×2	Bisphosphonate (not specified) 2 months ‐ oral	Denosumab 60 mg ×1
Biochemical changes^a^	↓Ca	↓Ca, ↑PTH, ALP	↓Ca, Pi ↑PTH, ALP	No change	↓Ca, Pi
Skeletal symptoms^a^	Not reported	↑ Disability & weakness	Not reported	No change	Not reported
Spinal BMD *T*‐score	−1.7	−2.4	Not reported	−4.5	−3.2
Other	Postgastric bypass	Postgastric bypass	Also had metastatic breast cancer		Myeloma

^a^ Indicates changes in symptoms and plasma biochemistry after administration of the antiresorptive agent.

ALP = alkaline phosphatase; Ca = calcium; F = female; M = male; Pi = phosphate; PTH = parathyroid hormone.

Long‐acting bisphosphonates are now very widely used in the treatment of osteoporosis and several other skeletal disorders. We emphasize that inhibitors of bone resorption must not be given to people with osteomalacia. In addition to biochemical changes, a marked deterioration in clinical state can occur; we suggest this because bone repair is inhibited. The effect may be prolonged with long‐acting agents such as zoledronate. It is important that clinicians consider the possibility of osteomalacia before attributing bone pain, fracture, and raised ALP levels to other causes. Simple biochemical tests of plasma calcium, phosphate, and ALP, if correctly interpreted, can alert clinicians to the possibility of hypophosphatasia, or calcipenic or phosphopenic osteomalacia. However, many clinical biochemistry panels currently available to physicians do not routinely include phosphate estimations: This too could contribute to missed diagnoses of phosphopenic osteomalacia. Bone scintigraphy has diagnostic value that is underappreciated.

## Disclosures

None of the authors have any conflict of interest.

## Peer Review

The peer review history for this article is available at https://publons.com/publon/10.1002/jbm4.10374.
